# The morphokinetics algorithm based on data from day 5 blastocyst transfer (KIDScoreD5 version 3) is also useful for embryo selection in day 6 blastocyst transfer

**DOI:** 10.1002/rmb2.12484

**Published:** 2022-09-13

**Authors:** Masashi Shioya, Tatsuya Kobayashi, Tomoharu Sugiura, Maki Fujita, Keiichi Takahashi

**Affiliations:** ^1^ Takahashi Women's Clinic Chiba Japan; ^2^ Department of Reproductive Medicine, Graduate School of Medicine Chiba University Chiba Japan

**Keywords:** blastocyst, embryo development, pregnancy outcome, single embryo transfer, time‐lapse imaging

## Abstract

**Purpose:**

To analyze whether the morphokinetics algorithm based on data from day 5 blastocyst transfer (KIDScoreD5 version 3) can predict the pregnancy rate of both day 5 and day 6 blastocyst transfers.

**Methods:**

The relationship between KIDScoreD5 and clinical pregnancy rate was evaluated using the Cochran–Armitage test and receiver‐operating characteristic (ROC) curve analysis.

**Results:**

A positive correlation was observed between the KIDScoreD5 value and clinical pregnancy rate for both day 5 (*p* = 0.0003) and day 6 blastocysts (*p* = 0.0019) using the Cochrane–Armitage test. ROC curve analysis showed that the area under the curve (AUC) of KIDScoreD5 for clinical pregnancy was 0.627 (0.575–0.677, *p* < 0.0001) for day 5 blastocysts and 0.685 (0.571–0.780, *p* = 0.0009) for day 6 blastocysts. The combined analysis of both day 5 and day 6 blastocysts also showed an AUC of 0.680 (0.636–0.720, *p* < 0.0001), suggesting that it is possible to select embryos that are more likely to result in pregnancy.

**Conclusions:**

KIDScoreD5 could predict pregnancy not only in day 5 blastocysts but also in day 6 blastocysts. When both day 5 and day 6 blastocysts are vitrified, embryo selection by KIDScoreD5 is possible with a high prediction ability of pregnancy.

## Graphical abstract

The morphokinetics algorithm (KIDScoreD5) was able to predict pregnancy not only in day 5 blastocyst transfer but also in day 6 blastocysts. ROC curve analysis showed that the area under the curve (AUC) of KIDScoreD5 for clinical pregnancy was 0.627 (0.575‐0.677, *p* < 0.0001) for day 5 blastocysts and 0.685 (0.571‐0.780, *p* = 0.0009) for day 6 blastocysts. The combined analysis of both day 5 and day 6 blastocysts also showed an AUC of 0.680 (0.636‐0.720, *p* < 0.0001), suggesting that it is possible to select embryos that are more likely to lead to pregnancy.

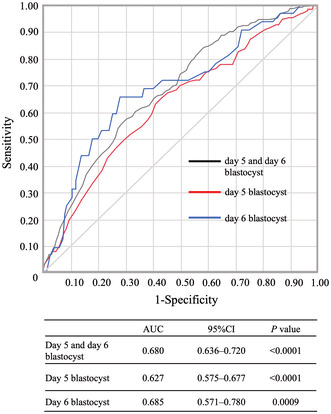

## 1 INTRODUCTION

Single embryo transfer avoids the risk of miscarriage and pregnancy complications associated with twin pregnancies.^1^, ^2^, ^3^ Therefore, the current trend in assisted reproductive technology (ART) is to achieve pregnancy with a minimum number of embryos to be used for transfer. Morphological evaluation is the most widely used selection criterion for blastocyst transfer.^4^ However, there are many cases in which blastocysts with suitable morphology do not result in pregnancy, suggesting that morphological evaluation is insufficient for reliably selecting embryos that result in pregnancy.^5^ Moreover, because morphological evaluation depends on the subjectivity of the observer, it is not consistent.^6^

One of the challenges of ART is to establish a simple and noninvasive method of embryo evaluation. Time‐lapse incubators allow for a noninvasive, nonsubjective, and detailed observation of the embryo developmental dynamics, and their usefulness has been reported. Embryos with abnormal cleavages, such as direct cleavage, are associated with a low pregnancy rate,^7^, ^8^ and embryo cleavage at the correct time is associated with transfer outcomes.^9^, ^10^ Observing the early stages of embryonic development may be useful for the prediction of pregnancy potential. In addition, the proper speed of development of blastocyst is also an important evaluation point. Embryos that develop into blastocysts on day 5 after fertilization are associated with a higher pregnancy rate than those that develop on day 6.^11^ Therefore, embryo selection based on morphokinetics (developmental dynamics and morphological evaluation) has been attempted and reported to improve clinical outcomes.^12^, ^13^ Previous reports have recommended the evaluation of such morphokinetics parameters using a time‐lapse incubator for embryo selection.^5^, ^14^, ^15^ In a monocentric ambispective study (prospective and retrospective) conducted by Boucret et al., abnormal embryo development detection by time‐lapse incubation was shown to improve embryo transfer outcomes.^5^

Embryoscope+ (Vitrolife, Gothenburg, Sweden), is a time‐lapse incubator that takes 11 focal plane pictures of culture embryos every 10 min and contains an algorithm, KIDScoreD5, for evaluating embryo morphokinetics. This algorithm is an embryo evaluation algorithm, which utilizes information from a large data set of known implantation data, comprehensively evaluates the developmental dynamics of the embryo, and performs morphological evaluation at the time of blastocyst formation, calculated in the form of a KIDScoreD5 value.^16^, ^17^, ^18^ KIDScoreD5 does not require daily observations for annotation. Embryos that have developed into blastocysts are semiautomatically calculated the time to each developmental stage and morphological grade by the guided annotation. After the observer agrees or makes slight modifications to each calculated parameter using guided annotation, the blastocysts are evaluated with a KIDScoreD5 value ranging from 1.0 to 9.9.

The first model of KIDScoreD5 (version 1) evaluated the fading time of the pronucleus, the timing of cleavage from zygotes toward the eight‐cell stage (t2, t3, t4, t5, and t8), the formation times of the blastocoele and blastocyst (tsB and tB), and the morphological grades of the inner cell mass (ICM) and trophectoderm (TE). In version 2, the timing of cleavage to the five‐cell stage (t2, t3, t4, and t5) and tB and TE morphology were evaluated. Both algorithms were formed using the data of 1100 transferred embryos. Reigner et al. reported that although both models predicted pregnancy, the value of the predictive ability was the same as that of traditional morphological evaluation (area under the curve [AUC] value = 0.60).^16^ The latest algorithm, version 3, was recently created using 5200 implanted embryos, and ICM, which was not considered in version 2, was added to the algorithm. A previous study demonstrated that the value of KIDScoreD5 (version 3) is a good predictor of pregnancy and live birth of transferred embryos^17^; however, in that study, day 5 and day 6 blastocysts were combined in the analysis. The KIDScoreD5 algorithm was based on day 5 blastocyst transfer, so it is unclear whether the transfer outcome of day 6 blastocysts transfer is also involved. Since a certain number of day 6 blastocyst transfers are also clinically performed, KIDScoreD5 must also be useful in selecting day 6 blastocysts. Therefore, the present study analyzed whether KIDScoreD5 (version 3) predicts the pregnancy rate of both day 5 and day 6 blastocyst transfers.

## 2 MATERIALS AND METHODS

### 2.1 Study design and population

This study was approved by the institutional review board of Takahashi Women's Clinic (protocol No. TWC20‐001). Consent was obtained in the form of an opt‐out through our clinic website and a bulletin board.

**FIGURE 1:**
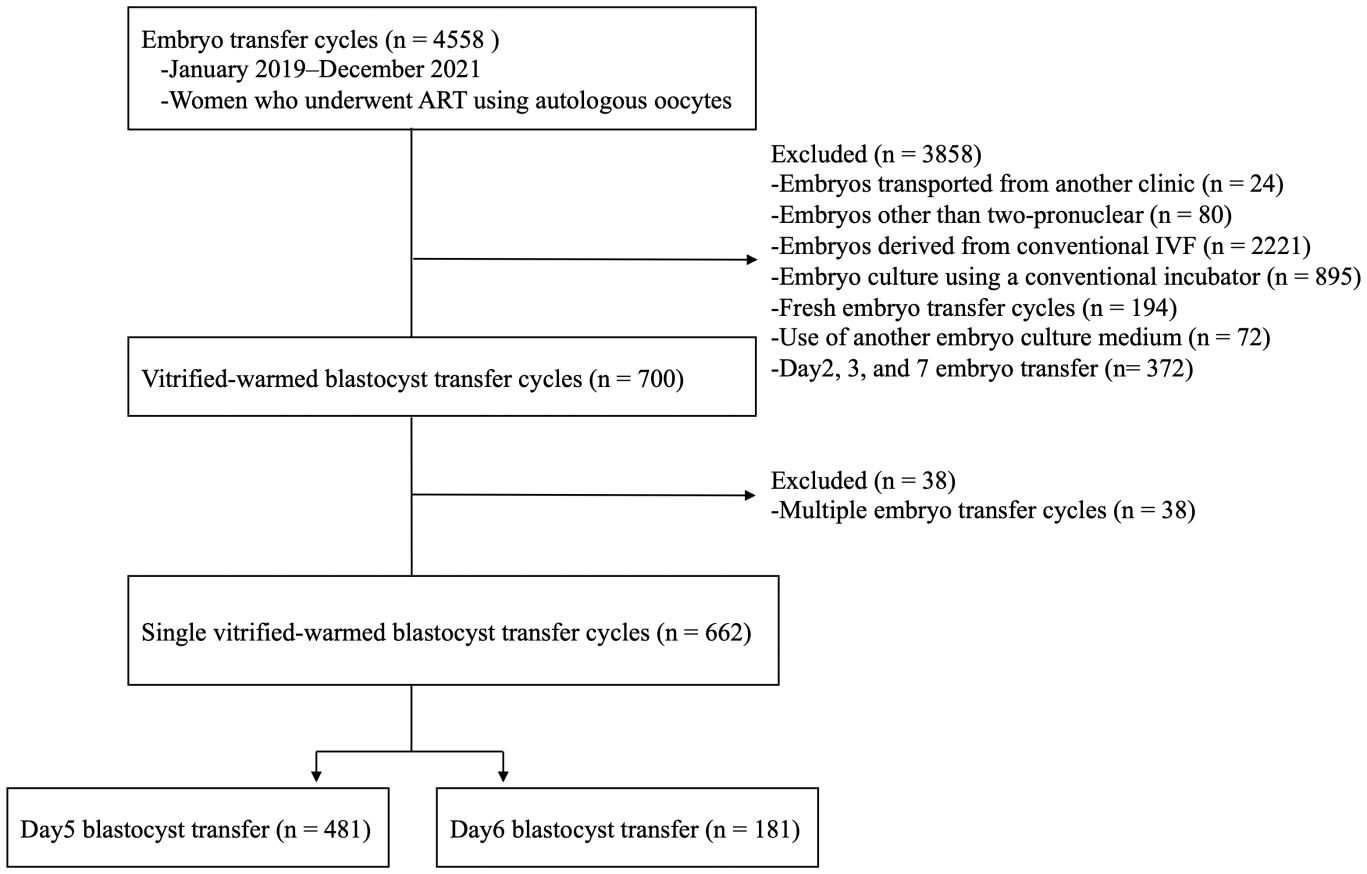
Flowchart for patient selection. PN, pronuclear

The inclusion criteria were: (1) women who underwent ART using autologous oocytes, (2) embryo transfer using day 5 and 6 blastocysts, and (3) single vitrified‐warmed blastocyst transfer cycles. The exclusion criteria were: (1) embryos transported from another clinic, (2) embryos other than two‐pronuclear embryos, (3) blastocysts derived from conventional in vitro fertilization (IVF), (4) embryo culture using a conventional incubator, (5) use of another embryo culture medium, and (6) multiple embryo transfer cycles (Figure 1).

### 2.2 Ovarian stimulation

Detailed ovarian stimulation protocols have been previously reported.^19^ All patients underwent ovarian stimulation based on serum anti‐Müllerian hormone (AMH) levels and follicle‐stimulating hormone (FSH) levels on day 3 of the menstrual cycle. Ovarian stimulation was performed using the mild stimulation protocol with clomiphene citrate (CC), gonadotropin‐releasing hormone (GnRH) antagonist protocol, or GnRH agonist protocol. Human chorionic gonadotropin and/or GnRH agonist was administered to induce ovulation when the leading follicle diameter reached 18 mm.

### 2.3 Oocyte retrieval and ICSI

Approximately 35–36 h after ovulation triggering, retrieval of oocytes was performed using a 20/17‐gauge needle (Vitrolife) under transvaginal ultrasound. Cumulus‐oocyte complexes were collected from the follicular fluid and transferred into an HEPES‐buffered medium (P + HEPES medium®; Naka Medical, Tokyo, Japan) under stereomicroscopy. Then, cumulus‐oocyte complexes were washed and precultured in an insemination medium (P + insemination medium®; Naka Medical) at 6.0% CO_2_, 5.0% O_2_, and 37.0°C for 1–3 h until ICSI. After preculture, oocytes were denuded from the surrounding cumulus cells in hyaluronidase (CooperSurgical, Trumbull, CT, USA), and oocytes with the first polar body were used for ICSI.

### 2.4 Embryo culture and evaluation

Embryo culture was performed using EmbryoScope+ (Vitrolife) with an EmbryoSlide+ culture dish (Vitrolife). After ICSI, oocytes were transferred into ONESTEP medium® (Naka Medical) droplets under OVOIL® (Vitrolife). We took 11 focal plane embryo images every 10 min to obtain the developmental dynamics of the embryos. Embryos showing two pronuclei were regarded as normally fertilized embryos and were cultured until the blastocyst stage up to 144 h at 37.0°C, 6.0% CO_2_, and 5.0% O_2_ concentration.

For KIDScoreD5 scoring, the developmental dynamics, ICM grade, and TE grade were evaluated semiautomatically (guided annotation) using EmbryoViewer® (Vitrolife). Developmental dynamics were evaluated as the time required for the development of two‐cell (t2), three‐cell (t3), four‐cell (t4), five‐cell (t5), and blastocyst (tB) stages. Morphological grades of ICM and TE were assigned as A, B, or C. Grade AA blastocysts were defined as an excellent grade, grades AB, BA, and BB as good grade, and grades AC, CA, BC, CB, and CC as poor grade. The KIDScoreD5 was calculated based on the observer's agreement with the developmental dynamics and morphological grades determined by EmbryoViewer®.

### 2.5 Embryo vitrification and single Vitrified‐Warmed blastocyst transfer

Blastocysts with more than 50% blastocoel formation on day 5 and day 6 were vitrified using the Vitrification Kit (Kitazato, Fuji, Japan) according to the manufacturer's protocol. In the spontaneous ovulatory cycles, endometrial thickness and ovulation were monitored in the patients using ultrasound. Estradiol (3 mg/day; Julina®, Bayer, Leverkusen, Germany) and dydrogesterone (30 mg/day; Duphaston®, Mylan Inc., Canonsburg, PA, USA) were orally administered when the endometrial thickness reached ≥8 mm and ovulation was confirmed. In the hormone replacement cycles, estradiol (1–3 mg/day) was administered daily beginning on days 3–5 of the menstrual cycle. Dydrogesterone (30 mg/day) was administered in addition to estradiol when the endometrium was confirmed to be ≥8 mm. In both embryo transfer cycles, embryo transfer was performed 5–6 days after initiating dydrogesterone administration. Vitrified blastocysts were used for single vitrified‐warmed blastocyst transfer in order of the highest grade based on Gardner's criteria. Embryos were warmed using the Thawing Kit (Kitazato) according to the manufacturer's protocol. After warming, embryos were cultured for 3–5 h until embryo transfer under the conditions of 37°C, 6% CO_2_, and 5% O_2_. All embryos were transferred using an embryo transfer catheter (Kitazato) under transabdominal ultrasound guidance. A clinical pregnancy was defined as an observation of a fetal heartbeat on transvaginal ultrasound guidance at 8 weeks after blastocyst transfer.

### 2.6 Statistical analysis

**FIGURE 2:**
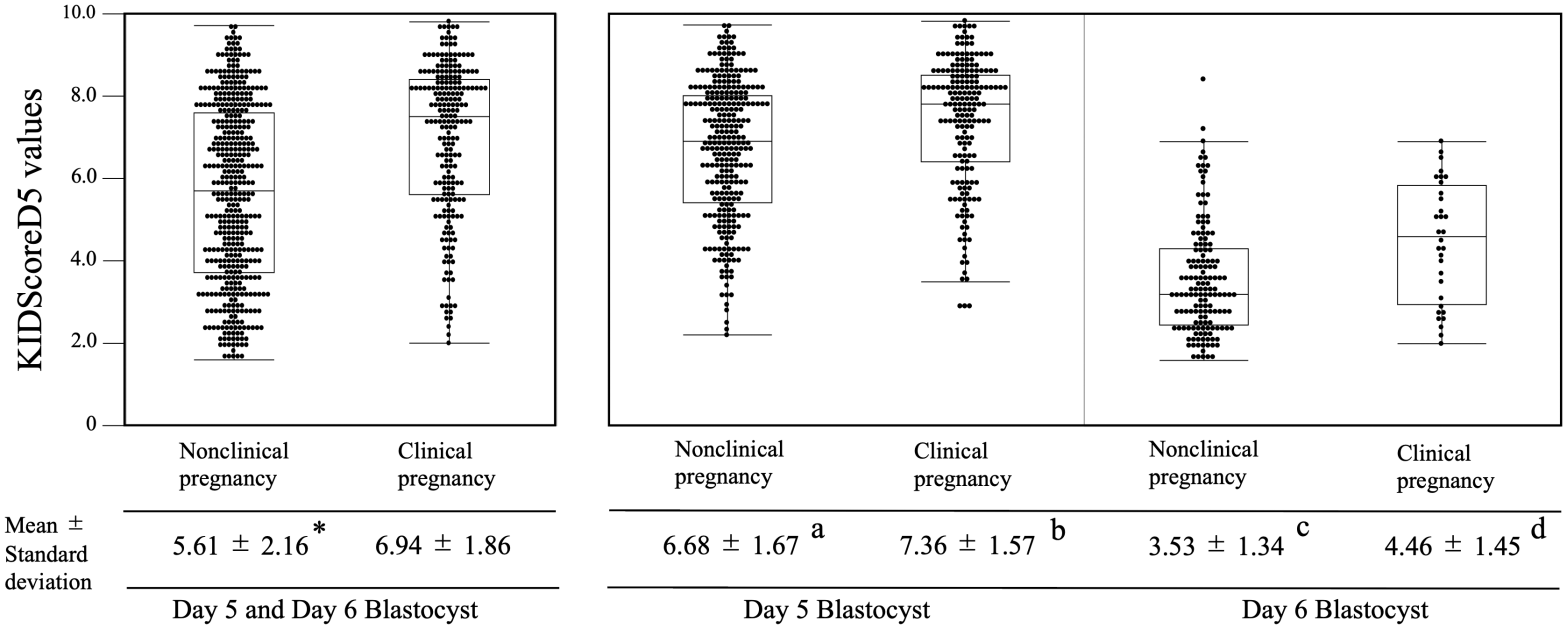
Comparison of the mean KIDScoreD5 values between the clinical pregnancy and nonclinical pregnancy blastocysts. Boxplots show the median, 25th/75th percentile, minimum, and maximum values. In the combined analysis of day 5 and day 6 blastocysts, the difference in the mean KIDScoreD5 values was compared using the Wilcoxon rank‐sum test, and a significant difference (*p < 0.0001) was noted between the presence and absence of pregnancy. In the separated analysis of day 5 and day 6 blastocysts, the mean KIDScoreD5 values were compared using Kruskal–Wallis test and Steel–Dwass test. Significant differences were noted between each group (different letters [a, b, c, d] indicate significant differences: *p* < 0.0001)

**FIGURE 3:**
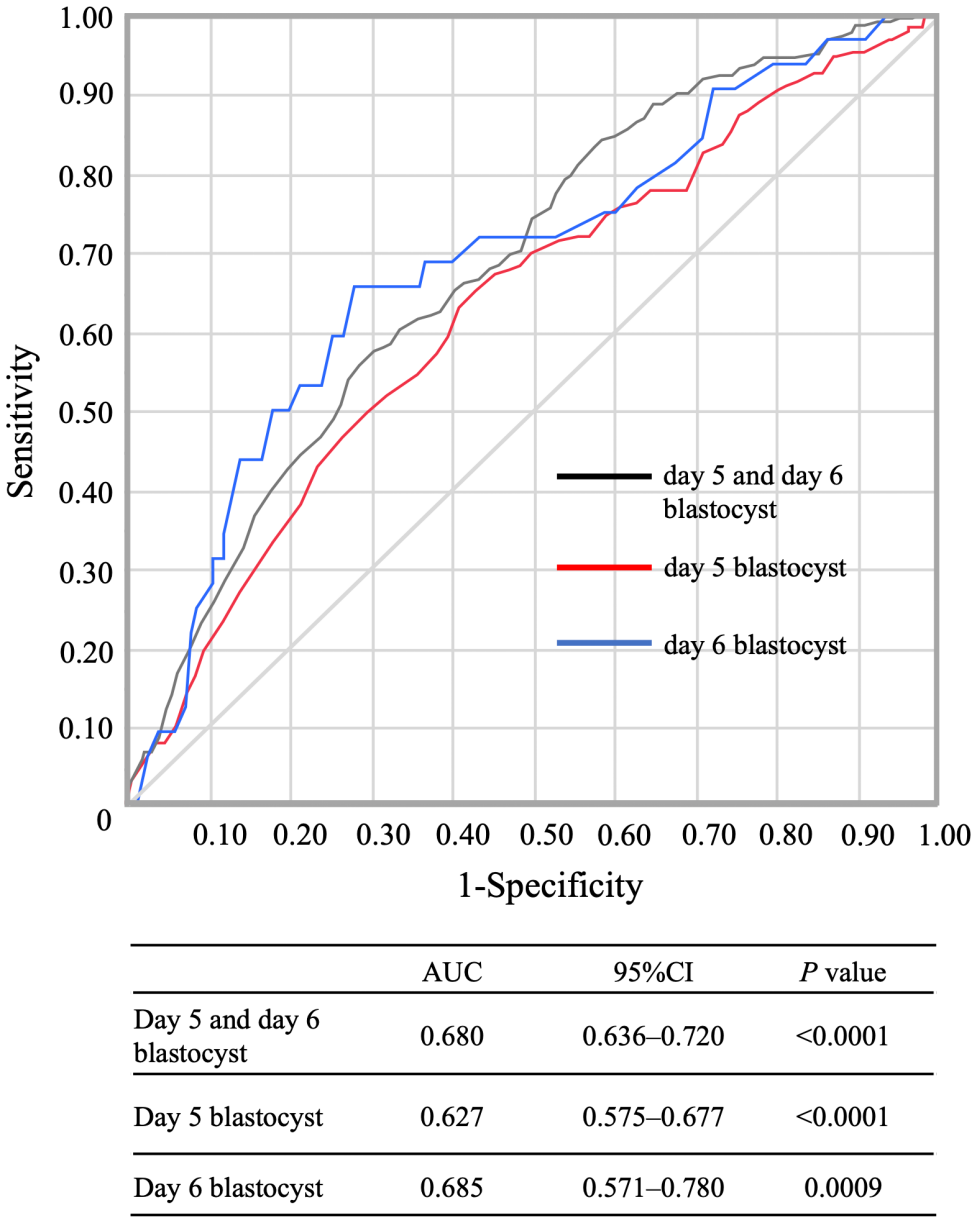
Evaluation of AUC values for clinical pregnancy predictive ability in the combined analysis (day 5 and 6) and the separated analysis (day 5 or 6). AUC values, 95% confidence intervals, and *p* values are shown

Statistical analysis was performed using JMP Pro 15.00 (SAS Institute, Cary, NC, USA). Categorical data were expressed as percentages and analyzed using the Chi‐square test or Fisher's exact test. Continuous data were expressed as mean and standard deviation and analyzed using the Wilcoxon rank‐sum test (Table 1). To compare the mean KIDScoreD5 values between embryos that resulted in clinical pregnancy and those that did not result in clinical pregnancy, we used the Wilcoxon rank‐sum test for the combined analysis of day 5 and day 6 blastocysts (Figure 2) and the Kruskal–Wallis and Steel–Dwass tests for the separated analysis of day 5 and day 6 blastocysts. Cochran–Armitage test was used to analyze the clinical pregnancy rate for each KIDScoreD5 value (Table 2). Multivariate logistic regression analysis using confounding variables (female age at blastocyst vitrified, body mass index, basal AMH level, and etiology of infertility) was used to analyze the relationship between KIDScoreD5 value and clinical pregnancy (Table 3). In the analysis of day 6 blastocysts, the number of transfer cycles was limited; therefore, a stepwise variable selection procedure was used to include variables with the best Akaike information criterion. To evaluate the predictive ability for a clinical pregnancy, a receiver‐operating characteristic (ROC) curve analysis with the KIDScoreD5 value and evaluated via AUC (Figure 3).

**TABLE 1:** Background of the patients and transferred blastocysts for the analysis

	Day 5 blastocyst	Day 6 blastocyst	*p* Value
No. of patients	291	132	
No. of blastocyst transfer cycles	481	181	
Female age at blastocyst vitrified (year)	36.27 ± 4.58	37.82 ± 4.50	<0.0001
Body mass index (kg/m^2^)	22.16 ± 4.04	22.20 ± 3.22	0.2300
Basal AMH (μg/L)	3.83 ± 3.42	2.60 ± 2.11	<0.0001
Basal FSH (μg/L)	6.73 ± 3.08	7.12 ± 2.35	0.0085
No. of previous embryo transfer cycles	4.21 ± 3.54	6.43 ± 4.49	<0.0001
Etiology of infertility (%)			
Male factor	16.01 (77/481)	8.84 (16/181)	0.0180
Tubal factor	2.29 (11/481)	2.76 (5/181)	0.7774
Ovarian disorders	10.60 (51/481)	9.94 (18/181)	0.8049
Uterine factor	8.73 (42/481)	12.71 (23/181)	0.1255
Multiple factors	32.22 (155/481)	38.12 (69/181)	0.1529
Other	30.15 (145/481)	27.62 (50/181)	0.5259
KIDScoreD5 value	6.95 ± 1.66	3.69 ± 1.40	<0.0001
Excellent‐grade blastocyst (AA)	61.95 (298/481)	14.92 (27/181)	<0.0001
Good‐grade blastocyst (AB, BA, and BB)	26.82 (129/481)	32.60 (59/181)	0.1417
Poor‐grade blastocyst (AC, CA, BC, CB, and CC)	11.23 (54/481)	52.49 (95/181)	<0.0001
Clinical pregnancy rate (presence of a fetal heartbeat)	39.29 (189/481)	17.68 (32/181)	<0.0001

*Note*: Results are shown as mean ± standard deviation or percentages when appropriate. BMI, basal AMH, and basal FSH are shown for female. Categorical data were analyzed by chi‐square test or Fisher's exact test, and other backgrounds were analyzed by Wilcoxon rank‐sum test.

Abbreviations: AMH, anti‐Müllerian hormone; FSH, follicle‐stimulating hormone.

**TABLE 2:** Clinical pregnancy rate for each KIDScoreD5 value

KIDScoreD5 value	1.0–1.9	2.0–2.9	3.0–3.9	4.0–4.9	5.0–5.9	6.0–6.9	7.0–7.9	8.0–8.9	9.0–9.9	*p* Value
Day 5 and day 6 blastocyst (%, *n*)	0 (0/9)	16.67 (11/66)	10.00 (7/70)	23.61 (17/72)	39.02 (32/82)	27.78 (25/90)	38.68 (41/106)	53.08 (69/130)	51.35 (19/37)	<0.0001
Day 5 blastocyst (%, *n*)	0 (0/0)	37.50 (3/8)	21.05 (4/19)	22.73 (10/44)	37.88 (25/66)	24.66 (18/73)	39.05 (41/105)	53.49 (69/129)	51.35 (19/37)	0.0003
Day 6 blastocyst (%, *n*)	0 (0/9)	13.79 (8/58)	5.88 (3/51)	25.00 (7/28)	43.75 (7/16)	41.18 (7/17)	0 (0/1)	0 (0/1)	0 (0/0)	0.0019

*Note*: Analysis was performed by the Cochrane–Armitage test. There was a significant trend between the KIDScoreD5 value and the clinical pregnancy rate in the day 5 and day 6 combined analysis and the day 5 or day 6 separated analysis.

**TABLE 3:** Multivariate logistic regression analysis considering patient backgrounds for clinical pregnancy

	Day 5 and day 6 blastocyst^a^			Day 5 blastocyst^a^			Day 6 blastocyst^b^		
	Adjusted OR	95% CI	*p* Value	Adjusted OR	95% CI	*p* Value	Adjusted OR	95% CI	*p* Value
Female age at blastocyst vitrified (year)	0.880	0.841–0.921	<0.0001	0.046	0.014–0.148	<0.0001	0.918	0.841–1.002	0.0579
Body mass index (kg/m^2^)	0.953	0.906–1.003	0.0631	0.255	0.069–0.936	0.0394	‐	‐	‐
Basal AMH (μg/L)	0.976	0.918–1.038	0.4388	0.983	0.919–1.050	0.6060	‐	‐	‐
Etiology of infertility									
Male factor	0.717	0.399–1.288	0.2655	0.620	0.327–1.178	0.1447	‐	‐	‐
Tubal factor	1.399	0.231–8.485	0.7153	1.665	0.229–12.127	0.6149	‐	‐	‐
Ovarian disorders	0.644	0.291–1.423	0.2765	0.491	0.200–1.208	0.1216	‐	‐	‐
Uterine factor	0.889	0.447–1.768	0.7373	0.981	0.444–2.167	0.9618	‐	‐	‐
Multiple factors	0.688	0.429–1.103	0.1206	0.627	0.367–1.072	0.0883	‐	‐	‐
KIDScoreD5	1.324	1.200–1.462	<0.0001	1.247	1.089–1.429	0.0014	1.566	1.194–2.053	0.0010

Abbreviations: AMH, anti‐Müllerian hormone; CI, confidence interval; OR, odds ratio.

^a^ Multivariate logistic regression analysis using confounding variables (female age at blastocyst vitrified, body mass index, basal AMH level, and etiology of infertility) was used to analyze the relationship between KIDScoreD5 value and clinical pregnancy.

^b^ A stepwise variable selection procedure was used to include variables with the best Akaike Information Criterion in the analysis of day 6 blastocysts because the number of transfer cycles was limited.

## 3 RESULTS

### 3.1 Characteristics of patients and embryos

Table 1 shows the background of the patients and blastocysts used for transfer. Patients who underwent ICSI between 2019 and 2021 were included in this study. A total of 423 patients underwent single vitrified‐warmed blastocyst transfer, 291 patients underwent day 5 blastocyst transfer, and 132 patients underwent day 6 blastocyst transfer. Female age at blastocyst vitrified (*p* < 0.0001), basal AMH level (*p* < 0.0001), basal FSH level (*p* = 0.0085), the number of previous embryo transfer cycles (*p* < 0.0001), and proportion of male factor infertility (*p* = 0.0180) were significantly different between the day 5 blastocyst group and the day 6 blastocyst group. No significant differences in body mass index (*p* = 0.2300) were observed between the groups. The day 5 blastocysts had a significantly higher mean KIDScoreD5 value (6.95 ± 1.66) than that of day 6 blastocysts (3.69 ± 1.40, *p* < 0.0001). The rates of excellent‐grade blastocysts (Gardner's criteria = AA) were 61.95% (298/481) and 14.92% (27/181), good‐grade blastocysts (Gardner's criteria = AB, BA, and BB) were 26.82% (129/481) and 32.60% (59/181), and poor‐grade blastocysts (Gardner's criteria = AC, CA, BC, CB, and CC) were 11.23% (54/481) and 52.49% (95/181) for day 5 and day 6 blastocysts, respectively. Day 5 blastocysts were associated with a significantly higher clinical pregnancy rate (32.29 vs. 17.68, *p* < 0.0001) than day 6 blastocysts.

### 3.2 Relationship between KIDScoreD5 values and clinical pregnancy rate

We evaluated the developmental dynamics and morphological grade using guided annotation, and the KIDScoreD5 values were assigned in a range of 1.0–9.9 points. After blastocyst transfer, the KIDScoreD5 values were compared with or without the presence of clinical pregnancy. For a combined analysis of day 5 and day 6 blastocysts, the mean KIDScoreD5 values of clinical pregnant blastocysts were significantly higher than those of nonclinical pregnant blastocysts (6.94 ± 1.86 vs. 5.61 ± 2.16, *p* < 0.0001; Figure 2). In analyzing day 5 and day 6 blastocysts separately, the mean KIDScoreD5 value was found to be higher in the clinical pregnant blastocysts than in the nonclinical pregnant blastocysts (day 5: 7.36 ± 1.57 vs. 6.68 ± 1.67, *p* < 0.0001; day 6: 4.46 ± 1.45 vs. 3.53 ± 1.34, *p* = 0.0010). However, the mean KIDScoreD5 values of day 5 nonclinical pregnant blastocysts were significantly higher than those of day 6 clinical pregnant blastocysts (6.68 ± 1.67 vs. 4.46 ± 1.45, *p* < 0.0001).

We analyzed the relationship between the clinical pregnancy rate and each 1.0 value of KIDScoreD5 using the Cochran–Armitage test (Table 2). A positive correlation was observed between KIDScoreD5 values and the pregnancy rate in the combined and separated analysis of day 5 and day 6 blastocysts.

Multivariate analysis showed that the KIDScoreD5 value affected the pregnancy outcome even when patient backgrounds and etiology of infertility were considered (Table 3).

### 3.3 Evaluation of KIDScoreD5 and clinical pregnancy using an ROC curve analysis

To determine whether KIDScoreD5 is accurate for predicting pregnancy, ROC curve analysis with KIDScoreD5 was performed (Figure 3). The AUC value was 0.680 (95% CI: 0.636–0.720, *p* < 0.0001) for the combined analysis of day 5 and day 6 blastocysts, 0.627 (95% CI: 0.575–0.677, *p* < 0.0001) for day 5 blastocysts, and 0.685 (95% CI: 0.571–0.780, *p* = 0.0009) for day 6 blastocysts. These results indicate that KIDScoreD5 can predict clinical pregnancy in both day 5 and day 6 blastocysts.

## 4 DISCUSSION

In some patients, the embryos develop slowly and only the day 6 blastocyst can be vitrified. In fact, 27.3% (181/662) of blastocyst transfers included in this study were day 6 blastocyst transfers. In addition, in a large cohort study by Kato et al., the KIDScoreD5 (version 3) of 2482 blastocysts was analyzed, of which 29.5% were day 6 blastocysts.^17^ In that study, day 5 and day 6 blastocysts were analyzed together. The KIDScoreD5 algorithm was created based on the results of day 5 blastocyst transfer, and it was not clear whether it could predict pregnancy rates for day 6 blastocyst transfer. Therefore, in the present study, we analyzed whether KIDScoreD5 has the ability to predict pregnancy not only in day 5 blastocyst transfer but also in day 6 blastocyst transfer.

We compared the relationship between KIDScoreD5 values and clinical pregnancy rate using the Cochrane–Armitage test and found that clinical pregnancy rate increased in proportion to the increase in KIDScoreD5 values for both day 5 and day 6 blastocysts. Furthermore, from the comparison of KIDScoreD5 values of blastocysts with or without clinical pregnancy, the KIDScoreD5 values were significantly higher in blastocysts with confirmed clinical pregnancy. This finding suggests that there is a positive correlation between KIDScoreD5 values and the clinical pregnancy rate in day 5 and day 6 blastocysts.

In recent years, preimplantation genetic testing for aneuploidies (PGT‐A) using next‐generation sequencing has enabled the analysis of the number of chromosomes^20^, ^21^ and the selection of embryos that are more likely to result in a live birth. Chromosomal status is the most important factor involved in pregnancy. However, PGT‐A is invasiveness since it requires a TE biopsy. If possible, an approach for the selection of embryos that is non‐invasive and easy is ideal. Therefore, it is desirable to establish a method for selecting embryos with higher pregnancy potential using a time‐lapse incubator to evaluate developmental dynamics and morphology. The proper speed and characteristics of embryonic development reportedly reflect the state of the chromosomes.^22^, ^23^, ^24^ Basile et al. analyzed early embryonic developmental dynamics and chromosome status by logistic regression analysis and reported that there were differences in t5 and t5‐t2 and CC3 developmental times between embryos with normal and abnormal chromosomes.^23^ Similarly, Lee et al. reported that the time to t5 and t8 is delayed, and the period of CC3 is prolonged in high‐frequency mosaic embryos.^24^ Thus, the progression of early embryo cleavage at the appropriate time reflects the state of the chromosomes and is a predictor of embryos likely to result in a successful pregnancy. Because the KIDScoreD5 algorithm evaluates this early embryonic development, our study found a high correlation between KIDScoreD5 and pregnancy rate.

To evaluate how well KIDScoreD5 predicts the clinical pregnancy rate, we performed an ROC curve analysis. As a result, the AUC value of day 5 and day 6 blastocysts was 0.627 (95% CI: 0.575–0.677, *p* < 0.0001) and 0.685 (95% CI: 0.571–0.780, *p* = 0.0009), respectively, indicating that KIDScoreD5 can predict clinical pregnancy. In addition, in the combined analysis of day 5 and day 6 blastocysts, a positive correlation between KIDScoreD5 and clinical pregnancy rate was observed. In the ROC curve analysis, the AUC was 0.680 (95% CI: 0.636–0.720, *p* < 0.0001), suggesting that KIDScoreD5 can be used to select embryos that are more likely to result in pregnancy, even in cycles in which both day 5 and day 6 blastocysts can be vitrified. The mean KIDScoreD5 value in the day 5 nonclinical pregnant group was significantly higher than that in the day 6 clinical pregnant group (Figure 2). Table 2 shows that day 6 blastocysts have a high clinical pregnancy rate for KIDScoreD5 values between 4.0 and 6.9. However, no significant difference in clinical pregnancy rate was found between day 5 and day 6 blastocysts within KIDScoreD5 values of 4.0–6.9 (28.96 [53/183] vs. 34.43 [21/61], *p* = 0.4214). In addition, the optimal cut‐off values based on the Youden index in the ROC curve analysis for clinical pregnancies were calculated to be 7.3 and 4.0 for day 5 and day 6 blastocysts, respectively. No significant difference was found in the clinical pregnancy rate between day 6 blastocysts above the cut‐off value (≥4.0) and day 5 blastocysts below the cut‐off value (<7.3) (33.33 [21/63] vs. 28.45 [66/232], *p* = 0.4508). These results indicate that, even though the KIDScoreD5 values for day 6 blastocysts tend to be lower, we consider it acceptable to transfer day 5 and 6 blastocysts according mainly to the KIDScoreD5 value.

In recent years, the widespread use of time‐lapse incubators has led to attempts to identify factors associated with pregnancy. Developmental kinetics analysis is expected to further improve the prediction of pregnancy rate in the future. Sciorio et al. used the EmbryoScope drawing tool to measure the diameter and maximum area of blastocysts and analyzed their involvement in pregnancy rates, and found that the median diameter of nonclinical pregnant blastocysts was significantly lower than that of clinical pregnant blastocysts.^25^ Furthermore, an increase in blastocyst maximum area of 1 μm resulted in a 2.6% increase in the odds ratio for pregnancy.^25^ Therefore, expansion of the blastocyst area is likely to be a significant predictor of pregnancy. Another study reported that contraction of the blastocyst has a negative effect on transfer outcomes.^26^ Blastocyst contraction and expansion is considered to cause thinning of the zona pellucida and promote hatching. However, a study by Niimura observed the amount of contraction in mouse embryos and reported that the hatching rate of embryos with strong contraction was lower than that of embryos with weak contraction.^27^ Later, another report concluded that the evaluation of embryo contraction was a factor that predicted the outcome of pregnancy in human embryos regardless of the morphological quality of the blastocyst.^28^ Because there has been a report of a higher number of contractions in aneuploid embryos,^29^ blastocyst contraction may be a factor in embryo selection for transfer. Although the mathematical details of calculating the KIDScoreD5 value have not been disclosed, future studies on the relationship between these embryo characteristics and pregnancy may help in designing a more accurate pregnancy prediction algorithm.

The limitation of this study was that it was a retrospective analysis conducted in a single IVF center. There are differences in culture methods among IVF centers, and it is expected that the AUC of KIDScoreD5 will differ among facilities. In addition, the data used in this study were obtained from the analysis of 662 blastocyst transfers, and only 181 blastocysts were included in the analysis of day 6 blastocysts. Because of the limited number of blastocysts used in this study, the number of calculations should be increased, and the AUC values may change if studies are conducted at other centers. Therefore, randomized controlled trials at multiple centers are required.

In conclusion, the findings of this study showed that the embryo evaluation algorithm constructed from the results of day 5 blastocyst transfer also had a high predictive ability for pregnancy in day 6 blastocyst transfer. Even among patients with both vitrified day 5 and day 6 blastocysts, KIDScoreD5 may assist in selecting embryos that are more likely to result in pregnancy.
